# Iowa State Veterinary Medical Association

**Published:** 1895-11

**Authors:** 


Secretary.

### IOWA STATE VETERINARY MEDICAL ASSOCIATION.

The eighth annual meeting convened at Des Moines, Iowa, at 10.30 A.M., September 9, 1895, with the President, Dr. J. Miller, of Ottumwa, in the chair.

The members answering to roll-call were: Drs. Stalker, Campbell, Miller, E. E. Sayers, Niles, G. A. Johnson, G. A. Scott, H. Shipley, S. H. Johnson, Besser, Bown, R. C. Sayers, Gibson, Brown, Simcoke, McBirney, Edwards, Whitbeck, L. U. Shipley, Austin, Replogle, Paine, Rich, Wheeler, Kingery, Walrod, Parslow, Hinkley, Fullerton, Drinkwater, Wake, and Geddes. Honorary members present: Drs. S. Stewart, Kansas City, Kansas; W. L. Williams, Bozeman, Mont.; T. S. Butler, Agricultural College, Mississippi, and C. A. Carey, Auborn, Ala. Visitors present were: Drs. James

Vincent, Shenandoah; J. L. Williamson, Jefferson; Chas. Williams, Northwood; H. E. Talbot, Des Moines; C. E. Stewart, Chariton; H. L. Stewart, Oakley; S. T. Miller, Shelby; D. H. Miller, Harlan; P. Malcolm, New Hampton; S. K. Hazlet, Elgin; A. Collasawitz, Dubuque; and Drs. L. Pearson, S. J. J. Harger, W. H. Hoskins, and T. B. Rayner, Philadelphia; J. C. Foelker, Allentown, Pa.; H. D. Gill, New York City; C. P. Lyman and F. H. Osgood, Boston; O. Schwartzkopf, Chicago; J. H. Wattles, Kansas City.

The minutes of the previous meeting were read and approved, after which President J. Miller addressed the convention:

"It is with pleasure I am privileged to open this morning our eighth annual meeting and to welcome so many of my professional associates, and to note the onward progress of our profession in the many fields that are opening after so many months of labor and study.

"While we are making some progress and our sphere of usefulness enlarging, yet we have much to do before we will have acquired the highest point of proficiency in the different branches of our profession. Our profession covers a large field, and so various are our duties in order to be efficient and skilful in every department the veterinarian of to-day has to be studious and industrious. More is expected of us than of our predecessors of even ten years ago.

"As your presiding officer there are many subjects of interest to which I should call your attention, but because of the brevity of this session, and the meeting of the United States Veterinary Medical Association which is to follow, I will bring before you only a few of the most important subjects for your consideration.

"The first topic both in interest and importance to us and the public at large is that of milk and dairy inspection. This question has been before us for some time, and while it is comparatively old to some of you, yet to others it is new, at least so far as any practical experience is concerned. There is no article of diet so universally used as milk, and none affords such a ready vehicle for carrying disease germs from the lower animals to man.

"It is difficult to impress upon councilmen, legislators, and some health officers, as well as the public at large, and the dairymen in particular, the importance of milk and dairy inspection. Some veterinarians have not kept themselves posted relative to the facts bearing upon this subject and the dangers attending it; consequently a few have to stem this great tide of ignorance and prejudice. Every veterinarian should be able to make an accurate physical and chemical, and, if need be, a microscopical test of milk. I believe that only qualified veterinarians should be appointed milk and dairy inspectors from a commercial as well as sanitary aspect.

"That condition of milk known as "stringy" or "ropy milk," which has been so prevalent in many localities during the past two years, causing considerable anxiety to the consumer and entailing no small loss to the producer, has been looked into some, but requires further investigation with a view to finding the cause and the best means of prevention. When we become efficient in every detail of this work, as well as good sanitarians, I believe the public will recognize our fitness for such positions. I predict that ere long competent veterinarians only will be appointed milk and dairy inspectors.

"It is quite generally known that tuberculosis is prevalent in this country, and that it has already been found to an alarming extent in different localities in this State. As the most of you know this is a contagious disease of a dangerous character, not only because it spreads from one member of a herd to others, causing considerable financial loss, but that it can, and has been known to be communicated through the milk-supply, and may be by the meat (if not well cooked), to the human family. Its progress is slow but sure in this and other States, and it is impossible to detect it by a physical examination in the incipient stage,—only by the tuberculin test. Thanks to that able German scientist, Koch, for giving to us such an accurate and valuable diagnostic agent! It is worthy of the search of a man's lifetime. I have had some practical experience with this of late in the testing of about three hundred and fifty cows under the supervision of Dr. J. McBirney, one of the assistant State veterinary surgeons. Of its reliability I am firmly convinced. Every veterinary surgeon should familiarize himself with the details of this work and insist that local boards of health shall require all dairy cows to be subjected to the tuberculin-test in order that we may early detect the disease and stamp it out.

"Another topic is that of meat inspection. It was not until the people began to suffer from disease, and the loss of their loved ones, and became acquainted with the fact that it was caused by the eating of unwholesome and diseased meats, that they awoke to the necessity of meat inspection. In a land where diseases of food-producing animals are so prevalent, and where such food enters largely into the diet of the people, it is of paramount importance that it be pure. Our population is increasing so rapidly, together with our increased export trade, that the demand for meats has increased and the price of meats has advanced; thus there is more inducement now than formerly to market animals of poor quality and actually diseased and injured.

"Anyone having a knowledge of this subject would have to observe but a short time the slaughter of animals at a large packing establishment without being convinced of the partial or total unfitness of many of the carcasses for consumption. I feel grateful to those of our professional legislators and others who have been instrumental in securing for us our present laws and regulations relative to this question. This enterprise is so far-reaching in its effects that it has gone slowly, and even now is only in its incipiency, and is only found in some of the large packing centres.

"It is only within the last six months that our Secretary of Agriculture has extended this to all slaughtering establishments having interstate trade, as you will see by amendment of March 2, 1895. However, this does not reach all of the towns and cities in our State, and it remains with us to agitate this question and by using our influence with councilmen, health officers, and legislators until this system is perfected. Every member of this Association should use his influence to this end in order that all our people should enjoy the same protection against an injurious meat-supply. We are only few in number, but by diligence and activity we can do much along this line, and thus be of use in protecting the health and happiness of our people and not in the least detract from our own welfare.

"We have now about one hundred and eighty graduated veterinarians in this State, a number commensurate with the demands for our professional services; and it does seem to me that the time has come for us to expect and insist upon some protecting measures. I believe all of the prominent professions, excepting the veterinary profession, have laws protecting their members from imposters infringing upon their rights by assuming their title and privileges and deceiving the public thereby. This is not just to us, and at the same time exposes the public to deception and fraud, and neither have any redress as the culprit goes free. Other States have recognized the desirableness of such protection, and I believe ours will if we will just present it in the right light and insist upon some action.

"There has been considerable loss sustained by stock-raisers in this and other States by a malady known as "corn-stalk disease." The real cause of this disease is yet a mystery, and we can do some valuable work in the interest of our profession by taking every possible pains in solving this mystery. It would be well if we use all diligence when opportunity presents itself to discover the cause by going into every detail, making culture-tests, holding post-mortems whenever possible, and observing closely pathological lesions.

"In conclusion I want to thank those of you who have done so much toward the progress of our work and the success of our meetings. Knowing something of the mutual benefits derived from our annual meeting, I trust this will prove no exception in this respect to those we have held in the past."

Discussions followed regarding stringy milk, tuberculosis and *modus operandi* of tuberculin-tests, and were participated in by Drs. Stuart, Miller, Walrod, Gibson, Vincent, Rich, McBirney, and Campbell.

Communications from several members of the State Board of Health and from the State Dairy Commissions were read by the Secretary. A communication from the Secretary of the Illinois State Veterinary Medical Association, relative to forming an Association for the States of the Mississippi Valley, was read and discussed. A vote was passed, upon motion of Dr. Gibson, duly seconded, to discourage the formation of the same, on the ground that it would detract from the interest of our members in the State and National Associations.

On motion of Dr. Stewart, duly seconded, a vote was passed to accept the resignations of Drs. J. E. Harrison, Burlington; W. R. Cooper, Ouasquiton; and H. M. Replogle, Centerville.

Discussion on the advisabillty of sending a delegate from the Society to the annual meeting of the American Public Health Association, to be held in Denver, Col., October 1–4, 1895, was taken part in by Drs. Miller, Kingery, McBirney, Gibson, and Stewart. On motion of Dr, Rich, seconded by Dr. Kingery, a vote was passed making Dr. Stalker delegate to that meeting, with Dr. W. B. Niles, alternate. Meeting adjourned until 1.30 P.M.

(To be continued.)

---

